# Activation of Autophagy by Low-Dose Silica Nanoparticles Enhances Testosterone Secretion in Leydig Cells

**DOI:** 10.3390/ijms23063104

**Published:** 2022-03-13

**Authors:** Jinlong Zhang, Rongrong Ye, Jason William Grunberger, Jiaqi Jin, Qianru Zhang, Raziye Mohammadpour, Nitish Khurana, Xianyu Xu, Hamidreza Ghandehari, Fenglei Chen

**Affiliations:** 1 College of Veterinary Medicine, Yangzhou University, Yangzhou 225009, China; zjl@yzu.edu.cn (J.Z.); yrr971106@163.com (R.Y.); savannahkini@163.com (J.J.); longlongzhang12345@126.com (Q.Z.); xuxianyu@yzu.edu.cn (X.X.); 2 Jiangsu Co-Innovation Center for Prevention and Control of Important Animal Infectious Diseases and Zoonoses, Yangzhou 225009, China; 3 Joint International Research Laboratory of Agriculture and Agri-Product Safety of the Ministry of Education of China, Yangzhou University, Yangzhou 225009, China; 4 Department of Pharmaceutics and Pharmaceutical Chemistry, University of Utah, Salt Lake City, UT 84112, USA; u1204188@utah.edu (J.W.G.); raziye.mohammadpour@utah.edu (R.M.); nitish.khurana@utah.edu (N.K.); hamid.ghandehari@utah.edu (H.G.); 5 Utah Center for Nanomedicine, University of Utah, Salt Lake City, UT 84112, USA; 6 Department of Biomedical Engineering, University of Utah, Salt Lake City, UT 84112, USA

**Keywords:** silica nanoparticles, testicular toxicity, autophagy, testosterone, Leydig cells, StAR

## Abstract

Silica nanoparticles (SNPs) can cause abnormal spermatogenesis in male reproductive toxicity. However, the toxicity and toxicological mechanisms of SNPs in testosterone synthesis and secretion in Leydig cells are not well known. Therefore, this study aimed to determine the effect and molecular mechanism of low doses of SNPs in testosterone production in Leydig cells. For this, mouse primary Leydig cells (PLCs) were exposed to 100 nm Stöber nonporous spherical SNPs. We observed significant accumulation of SNPs in the cytoplasm of PLCs via transmission electron microscopy (TEM). CCK-8 and flow cytometry assays confirmed that low doses (50 and 100 μg/mL) of SNPs had no significant effect on cell viability and apoptosis, whereas high doses (more than 200 μg/mL) decreased cell viability and increased cell apoptosis in PLCs. Monodansylcadaverine (MDC) staining showed that SNPs caused the significant accumulation of autophagosomes in the cytoplasm of PLCs. SNPs activated autophagy by upregulating microtubule-associated protein light chain 3 (LC3-II) and BCL-2-interacting protein (BECLIN-1) levels, in addition to downregulating sequestosome 1 (SQSTM1/P62) level at low doses. In addition, low doses of SNPs enhanced testosterone secretion and increased steroidogenic acute regulatory protein (StAR) expression. SNPs combined with rapamycin (RAP), an autophagy activator, enhanced testosterone production and increased StAR expression, whereas SNPs combined with 3-methyladenine (3-MA) and chloroquine (CQ), autophagy inhibitors, had an opposite effect. Furthermore, BECLIN-1 depletion inhibited testosterone production and StAR expression. Altogether, our results demonstrate that low doses of SNPs enhanced testosterone secretion via the activation of autophagy in PLCs.

## 1. Introduction

Silica nanoparticles (SNPs), as one of the five main nanomaterials classified by the Consumer Products Inventory (CPI), are extensively used in consumer products and biomedical research [1]. Humans and animals are inevitably exposed to SNPs intentionally or unintentionally through oral, inhalation, or skin exposure [2,3]. Because of their small size and large surface area, SNPs can exhibit unique bioactivities and interactions with cellular or subcellular structures [4,5]. Although extensive efforts have been made to evaluate the possible toxicity and the underlying toxicological mechanisms of SNPs, such studies have not been thorough and systematic, especially for reproductive toxicity. It is well known that fertility and successful reproduction are essential to sustain a species. Therefore, SNP-induced reproductive toxicity should be investigated to increase public awareness.

In males, testes are vulnerable and sensitive to exposure to numerous nanoparticles, such as carbon black nanoparticles (CBNPs) and SNPs [6,7,8,9,10]. SNPs have been observed to pass through the blood–testis barrier and distribute inside testicular tissue [11,12,13]. Researchers have shown that SNPs do not significantly cause histological abnormalities or reversible damage in the testes; however, they decreased the daily number of sperm and fetus per clutch and increased the sensibility to oxidative stress [13]. Xu et al. and Ren et al. demonstrated that 105 nm of non-porous spherical SNPs (2 and 20 mg/kg) inhibited the spermatogenesis process via oxidative stress in vivo [12,14]. They also reported 93 nm of non-porous spherical SNPs (5 μg/mL) induced cytotoxicity in mice spermatocyte cells in vitro [15]. Although many studies have focused on spermatogenesis in male reproductive toxicity, the toxicity and toxicological mechanisms of SNPs are often overlooked in Leydig cells.

It is well known that the critical roles of Leydig cells are to synthesize and secrete testosterone, which contributes to spermatogenesis, promotes male sexual differentiation, and maintains male secondary sex characteristics [16]. Previous studies have demonstrated that autophagy is activated in Leydig cells and participates in testosterone production [17,18]. Meanwhile, a growing body of literature suggests that SNPs can activate autophagy or induce autophagy dysfunction [15,19,20,21,22,23,24,25,26]. However, there is no literature on the correlation between SNP-activated autophagy with testosterone synthesis in Leydig cells. Therefore, to verify whether SNP-induced testicular toxicity is related to Leydig cell dysfunction and the cooperation of autophagy in this process, we aimed to determine the role and molecular mechanism of autophagy in low doses of SNP-regulated testosterone synthesis and secretion in mouse Leydig cells, which is important to fully understand the toxicity of SNPs in the male reproductive system.

## 2. Results

### 2.1. Characterization of SNPs

**Figure 1:**
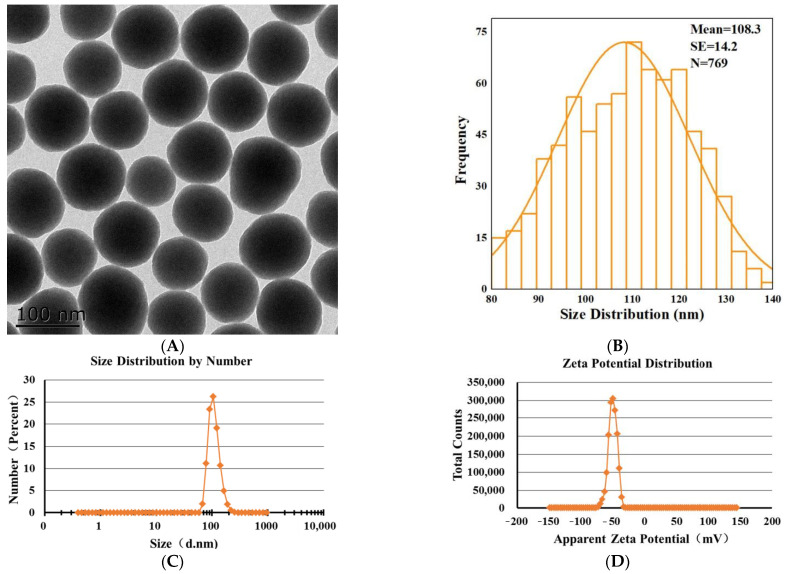
Characterization of SNPs. (**A**) TEM image of SNPs. Scale bar, 100 nm. (**B**) Analysis of the size distribution via Image J software. The average diameter of SNPs was 108.3 ± 14.2 nm. (**C**) The hydrodynamic diameter of SNPs was 110.8 ± 26.5 nm, with a PDI of 0.02 in 10 mM NaCl. (**D**) The apparent zeta potential of SNPs was −49.7 ± 6.6 mV in 10 mM NaCl.

Transmission electron microscopy (TEM) image showed that SNPs were spherical with low polydispersity (Figure 1A). The size distribution indicated that the average diameter of the particles was 108.3 ± 14.2 nm (Figure 1B). The representative hydrodynamic diameter of SNPs in 10 mM NaCl, following the NIST-NCL protocol [27], was 110.8 ± 26.5 nm, with a PDI of 0.02 (Figure 1C). The zeta potential of SNPs measured in 10 mM NaCl, following the NCL protocol [28], was −49.7 ± 6.6 mV (Figure 1D).

### 2.2. Cellular Internalization of SNPs

**Figure 2:**
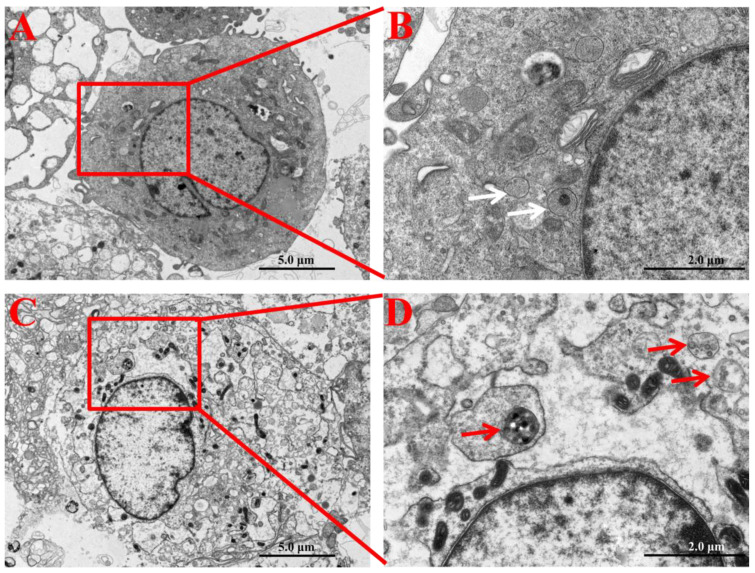
Cellular internalization and localization of SNPs in PLCs. (**A**) TEM image of PLCs in the control group (without SNP exposure). (**B**) Zoomed in image of the red box marked in **A**. White arrows indicate that vesicles did not include SNPs in granulosa cells. (**C**) TEM image of PLCs exposed to 100 µg/mL SNPs. (**D**) Zoomed in image of the red box in **C**. Red arrows indicate that vesicles enclosed SNPs in the cytoplasm of granulosa cells. Scale bar, 5.0 µm (**A**,**C**) and 2.0 µm (**B**,**D**).

In order to ensure the cellular internalization and localization of SNPs in PLCs, their intracellular distribution was determined by TEM. Compared to the control group (Figure 2A,B), PLCs demonstrated accumulation of particles in the cytoplasm of granulosa cells in the SNP-exposed group (Figure 2C). The enlarged image shows that SNPs were located in membranous vesicles of the cytoplasm (Figure 2D).

### 2.3. Effect of SNPs on Cell Viability and Apoptosis in PLCs

**Figure 3:**
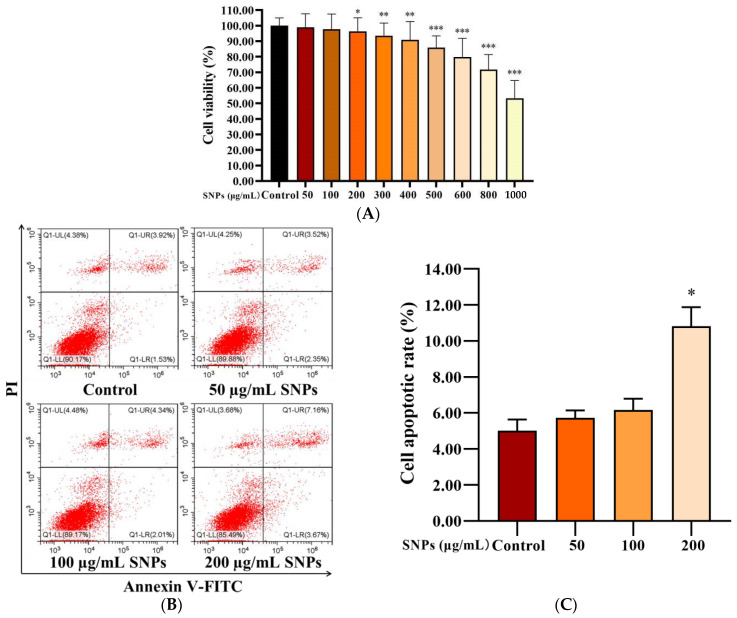
Effect of SNPs on cell viability in PLCs. (**A**) CCK-8 assay of the effect of SNPs on cell viability in PLCs. Cells were exposed to 0, 50, 100, 200, 300, 400, 500, 600, 800, and 1000 µg/mL SNPs for 24 h. (**B**) Flow cytometry analysis of the effect of SNPs on cell apoptosis in PLCs. After exposure to 0, 50, 100, and 200 µg/mL SNPs for 24 h, PLCs were collected for annexin V-FITC/PI staining. The UL quadrant indicates cell death caused by mechanical damage or necrotic cells, the UR quadrant indicates late apoptotic cells, the LL quadrant indicates normal cells, and the LR quadrant indicates early apoptotic cells. (**C**) The quantification of cell apoptosis in **B** is shown in the bar graphs. Data are presented as the mean ± SEM of three independent experiments. * Statistically different from the control (* *p* < 0.05, ** *p* < 0.01, and *** *p* < 0.001).

PLCs were exposed to 0–1000 µg/mL SNPs for 24 h to determine cell viability via CCK-8 assay. Low doses of SNPs (0–100 µg/mL) did not significantly affect cell viability, whereas high doses of SNPs (200–1000 µg/mL) significantly decreased cell viability (Figure 3A). The morphology of PLCs was observed; loss in the normal morphology started gradually and appeared at 200 µg/mL of SNP treatment (Figure S1). Consistent with the results of the CCK-8 assay, flow cytometry analysis showed that the apoptotic rate had no significant difference in the 50 and 100 µg/mL SNP-exposed groups [(5.72 ± 0.41)% and (6.16 ± 0.63)%, respectively], whereas significantly increased cell apoptosis in the 200 µg/mL SNP-exposed group (10.81 ± 1.07)% was observed compared to the control group (5.00 ± 0.63)% (Figure 3B,C).

### 2.4. SNPs Activated Autophagy in PLCs

**Figure 4:**
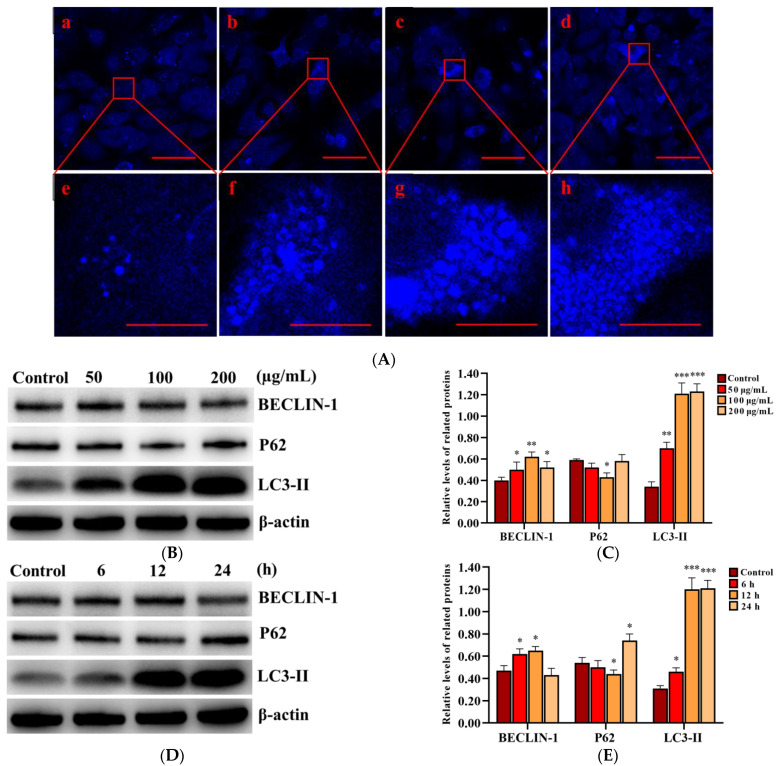
Effect of SNPs on the activation of autophagy in PLCs. (**A**) Fluorescence images of MDC staining. PLCs were exposed to 0, 50, 100, and 200 µg/mL SNPs for 12 h. Scale bar: 50 µm (**A**a–d) and 10 µm (**A**e–h). (**B**) Western bolt analysis of BECLIN-1, P62, and LC3-II levels. PLCs were exposed to 0, 50, 100, and 200 µg/mL SNPs for 12 h. (**C**) Quantification of the band intensity in **B** is presented as the relative ratio of target proteins to β-actin. (**D**) Western bolt analysis of BECLIN-1, P62, and LC3-II levels. PLCs were exposed to 100 µg/mL SNPs for 0, 6, 12, and 24 h. (**E**) Quantification of the band intensity in **D** is presented as the relative ratio of target proteins to β-actin. Data are presented as the mean ± SEM of three independent experiments. * Statistically different from the control is marked with asterisks (* *p* < 0.05, ** *p* < 0.01, and *** *p* < 0.001).

In order to determine the activation of autophagy, MDC staining was performed to detect autophagic vacuoles in PLCs after exposure to 0, 50, 100, and 200 µg/mL SNPs for 12 h. The fluorescence intensity in the SNP-exposed groups was higher than that of the control group (Figure 4A). MDC-labeled puncta in the SNP-exposed groups were also dose-dependently higher than those in the control group (Figure 4A). Western blot analysis showed that BECLIN-1 was significantly increased in the different groups exposed to a dose for 12 h (Figure 4B,C) and the group exposed to 100 µg/mL SNPs for 6 and 12 h (Figure 4D,E), whereas there were no significant differences with the group exposed to 100 µg/mL SNPs for 24 h (Figure 4D,E). P62 exhibited a decrease in the group exposed to 50 µg/mL SNPs for 12 h (Figure 4B,C) and the group exposed to 100 µg/mL SNPs for 6 h (Figure 4D,E) and was significantly decreased in the group exposed to 100 µg/mL SNPs for 12 h (Figure 4B–E). However, there was a significant increase in the group exposed to 100 µg/mL SNPs for 24 h (Figure 4D,E). LC3-II expression was significantly increased in the different dose-exposed and time-exposed groups compared to the control group (Figure 4B–E).

### 2.5. SNPs Enhanced Testosterone Secretion in PLCs

**Figure 5:**
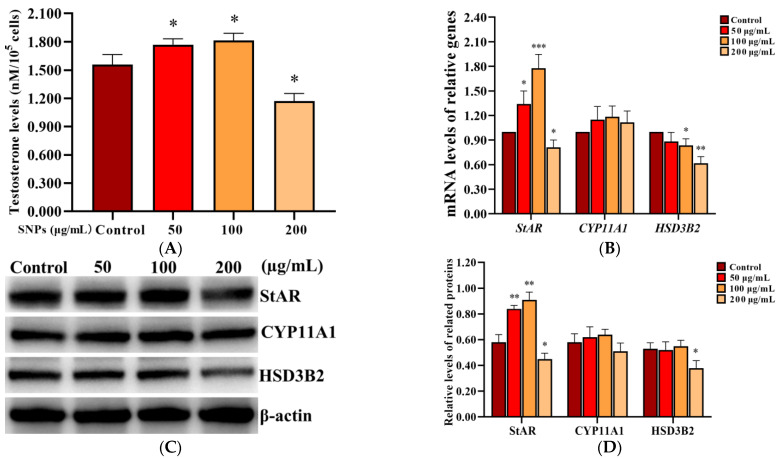
Effect of SNPs on the secretion of testosterone in PLCs. (**A**) Detection of the concentration of testosterone via ELISA analysis in the PLC culture supernatant. (**B**) Measurement of *StAR*, *CYP11A1*, and *HSD3B2* mRNA levels in PLCs via qRCR analysis. (**C**) Determination of the levels of StAR, CYP11A1, and HSD3B2 proteins in PLCs via western blot analysis. PLCs were exposed to 0, 50, 100, and 200 µg/mL SNPs for 12 h, respectively. (**D**) Quantification of the band intensity in **C** was presented by the relative ratio of target proteins to β-actin. Data are presented as the mean ± SEM of three independent experiments. * Statistically different from the control (* *p* < 0.05, ** *p* < 0.01, and *** *p* < 0.001).

To assess the effect of SNPs on testosterone secretion, the level of testosterone was measured in the cell culture supernatant in PLCs after exposure to 0, 50, 100, and 200 µg/mL SNPs for 12 h, via ELISA analysis. The concentration of testosterone significantly increased in the 50 and 100 µg/mL SNP-exposed groups, while significantly decreased in the 200 µg/mL SNP-exposed group compared to the control (Figure 5A). Furthermore, the mRNA levels of steroidogenic acute regulatory protein (*StAR*), cholesterol side-chain cleavage enzyme (*CYP11A1*), and *HSD3B2* were detected via qRCR analysis. *StAR* mRNA level was significantly increased in the 50 and 100 µg/mL SNP-exposed groups and significantly decreased in the 200 µg/mL SNP-exposed group compared to the control group (Figure 5B). *CYP11A1* mRNA level had no significant difference among the SNP-exposed groups compared to the control group (Figure 5B). *HSD3B2* mRNA level was significantly decreased in the 100 and 200 µg/mL SNP-exposed groups, while there were no significant difference in the 50 µg/mL SNP-exposed group compared to the control group (Figure 5B). Western blot analysis showed that StAR protein level was significantly increased in the 50 and the 100 µg/mL SNP-exposed groups, while significantly decreased in the 200 µg/mL SNP-exposed group compared to the control group (Figure 5C,D). CYP11A1 had no significant difference among the SNP-exposed groups compared to the control group (Figure 5C,D). HSD3B2 was significantly decreased in the 200 µg/mL SNP-exposed group compared to the control group (Figure 5C,D).

### 2.6. SNP-Activated Autophagy Enhanced Testosterone Secretion in PLCs

**Figure 6:**
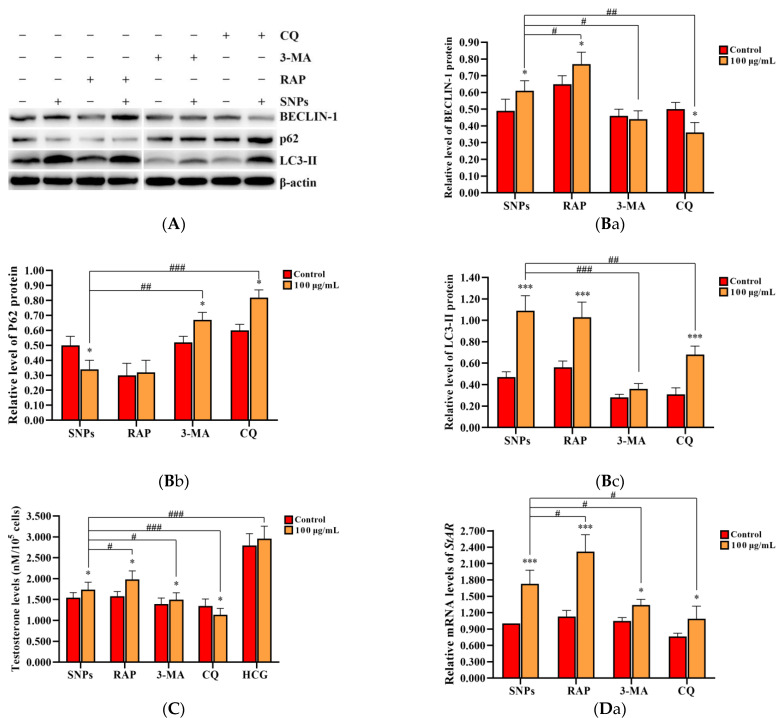

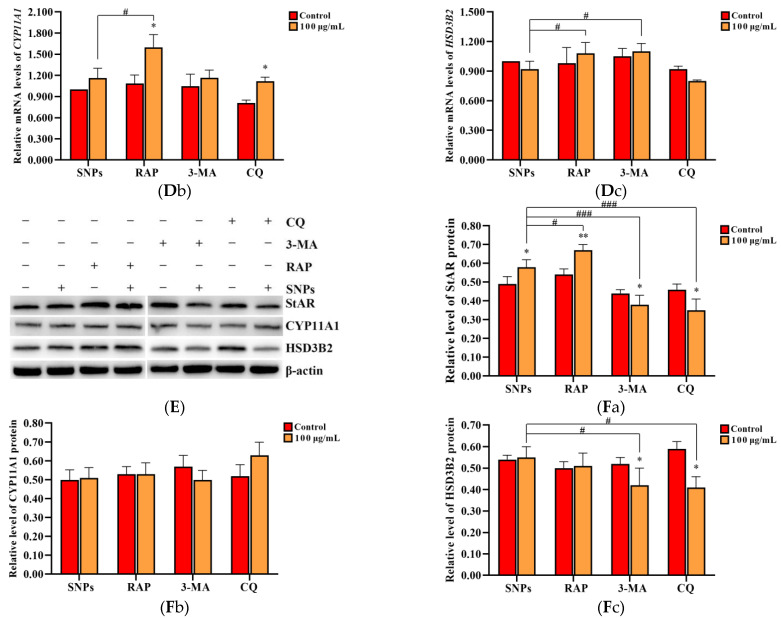
Effect of SNP-activated autophagy on testosterone secretion in PLCs. (**A**) Determination of BECLIN-1, P62, and LC3-II protein levels in PLCs via Western blot analysis. (**B**) Quantification of the band intensity in **A** is presented as the relative ratio of BECLIN-1 (**B**a), P62 (**B**b), and LC3-II (**B**c) to β-actin. (**C**) Detection of the concentration of testosterone via ELISA analysis in the PLC culture supernatant. (**D**) Measurement of *StAR* (**D**a), *CYP11A1* (**D**b), and *HSD3B2* (**D**c) mRNA levels in PLCs via qRCR analysis. (**E**) Determination of the levels of StAR, CYP11A1, and HSD3Β2 in PLCs via Western blot analysis. PLCs were exposed to 100 µg/mL SNPs for 12 h before exposure to 0.2 µM 3-MA, 5 µM CQ, and 1 µM RAP for 6 h. (**F**) Quantification of the band intensity in **E** is presented as the relative ratio of StAR (**F**a), CYP11A1 (**F**b), and HSD3Β2 (**F**c), respectively, to β-actin. Data are presented as the mean ± SEM of three independent experiments. * Statistically different from the control (* *p* < 0.05, ** *p* < 0.01, and *** *p* < 0.001); ^#^ statistically different from the SNP group (^#^ *p* < 0.05, ^##^ *p* < 0.01, and ^###^ *p* < 0.001).

To evaluate the effect of SNP-activated autophagy on testosterone secretion, PLCs were dosed with 100 µg/mL SNPs and pre-exposed to the autophagy activator RAP, as well as the inhibitors 3-MA and CQ. Western blot analysis showed that the BECLIN-1 protein level was significantly increased in the RAP + SNP-exposed group and significantly decreased in the 3-MA + SNP and the CQ + SNP-exposed groups compared to the SNP-only group (Figure 6A,Ba). P62 exhibited no significant difference in the RAP + SNP-exposed group and was significantly increased in the 3-MA + SNP and CQ + SNP-exposed groups compared to the SNP-only group (Figure 6A,Bb). LC3-II exhibited no significant difference in the RAP + SNP-exposed group and was significantly decreased in the 3-MA + SNP-exposed group and the CQ + SNP-exposed group compared to the SNP-only group (Figure 6A,Bc). ELISA analysis showed that the concentration of testosterone was significantly increased in the RAP + SNP-exposed group and significantly decreased in the 3-MA + SNP and the CQ + SNP-exposed groups compared to the SNP-only group (Figure 6C). qRCR analysis showed that *StAR* mRNA levels were significantly increased in the RAP + SNP-exposed group and significantly decreased in the 3-MA + SNP and the CQ + SNP-exposed groups compared to the SNP-only group (Figure 6Da). *CYP11A1* was significantly increased in the RAP + SNP-exposed group, with no significant difference in the 3-MA + SNP and the CQ + SNP-exposed groups compared to the SNP-only group (Figure 6Db). *HSD3B2* significantly increased in the RAP + SNP and the 3-MA + SNP-exposed groups and significantly decreased in the CQ + SNP-exposed group compared to the SNP-only group (Figure 6Dc). Western blot analysis showed that the StAR protein level was significantly increased in the RAP + SNP-exposed group and significantly decreased in the 3-MA + SNP and the CQ + SNP-exposed groups compared to the SNP-only group (Figure 6E,Fa). CYP11A1 exhibited no significant difference in all groups (Figure 6E,Fb). HSD3Β2 had no significant difference in the RAP + SNP-exposed group and was significantly decreased in the 3-MA + SNP and the CQ + SNP-exposed groups compared to the SNP-only group (Figure 6E,Fc).

### 2.7. BECLIN-1 Depletion Inhibited Testosterone Secretion in PLCs

**Figure 7:**
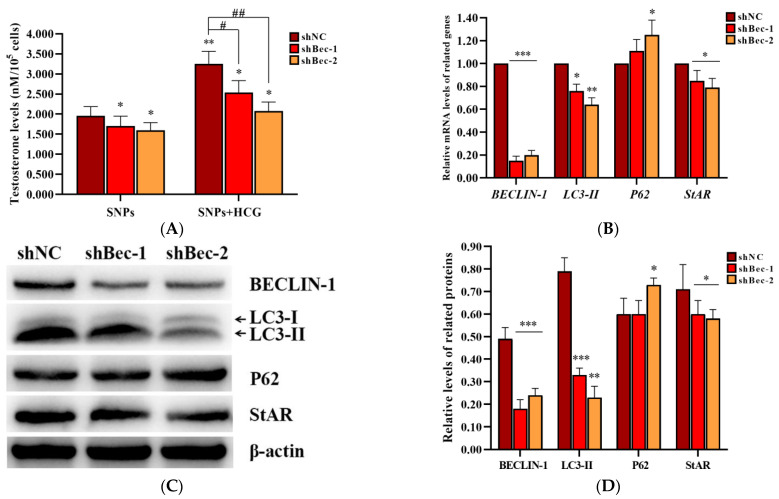
Effect of BECLIN-1 knockdown on testosterone secretion in PLCs. (**A**) Detection of the concentration of testosterone via ELISA analysis in the PLC culture supernatant. (**B**) Measurement of *BECLIN-1*, *LC3-II*, *P62*, and *StAR* mRNA levels in PLCs via qRCR analysis. (**C**) Determination of the expression of BECLIN-1, LC3-II, P62, and StAR protein levels in PLCs via Western blot analysis. PLCs were transduced with BECLIN-1 knockdown lentiviruses and exposed to 100 µg/mL SNPs in the absence or presence of 1 IU/mL HCG for 12 h. (**D**) Quantification of the band intensity in **C** is presented as the relative ratio of target proteins to β-actin. Data are presented as the mean ± SEM of three independent experiments. * Statistically different from the shNC + SNP group (* *p* < 0.05, ** *p* < 0.01, and *** *p* < 0.001); ^#^ statistically different from the shNC + SNP + HCG group (^#^ *p* < 0.05 and ^##^ *p* < 0.01).

To further demonstrate the role of SNP-activated autophagy in testosterone secretion, BECLIN-1 shRNA lentivirus vectors were constructed, and the packaged lentiviruses were transduced into PLCs. ELISA analysis showed that the concentration of testosterone significantly decreased in both shBec-1 + SNP and shBec-2 + SNP groups compared to the shNC + SNP group; furthermore, it was also significantly decreased after exposed to human chorionic gonadotropin (HCG) (Figure 7A). qRCR analysis showed that *BECLIN-1*, *LC3-II*, and *StAR* mRNA levels were significantly decreased, whereas *P62* mRNA levels were significantly increased in both shBec-1 + SNP and shBec-2 + SNP groups compared to the shNC + SNP group (Figure 7B). Western blot analysis showed that BECLIN-1, LC3-II, and StAR protein levels were all significantly decreased in both shBec-1 + SNP and shBec-2 + SNP groups, whereas P62 exhibited no significant difference in the shBec-1 + SNP group and was significantly increased in the shBec-2 + SNP group compared to the shNC + SNP group (Figure 7C,D).

## 3. Discussion

An increasing number of studies is focusing on the male reproductive toxicity of SNPs [6,13,15,29,30]. SNPs can penetrate the mouse blood–testis barrier and exert their toxic actions. After intravenous [12], intramuscular [31], or inhalation [13] exposure, SNPs have been observed to accumulate in the testes, impairing spermatogenesis and decreasing sperm quality and quantity. The testicular toxicity of SNPs can affect spermatogenesis, as well as testosterone level. Leydig cells are one of the important components of the testes and mainly participate in testosterone synthesis and secretion [32]. However, less attention is paid to the toxicity of Leydig cells after SNP exposure. In the current study, we demonstrate that low doses of SNPs can activate autophagy and enhance testosterone secretion in mouse Leydig cells.

The toxic effect of SNPs on PLCs directly affects the development of the male reproductive system. Not surprisingly, abnormally high doses of SNPs reduced cell viability and increased cell apoptosis [30]. Although low doses of SNPs have no significant cytotoxicity in PLCs, their effect on testosterone synthesis and secretion is not known. Nanoparticle-rich diesel exhaust (NR-DE, 149.0 ± 8.0 µg/m^3^) significantly increased the plasma or testicular testosterone levels and StAR levels in male rats [33]. A low dose (50 mg/kg) of zinc oxide nanoparticles (ZnO NPs) significantly increased serum testosterone concentration and the expression of *StAR* genes [30]. Furthermore, a low dose (3.0 mg/kg) of ZnO NPs significantly increased serum testosterone levels and protected against doxorubicin-induced testicular toxicity and DNA damage in male rats [34]. Long, rod-shaped mesoporous SNPs (9.5 nm pore size) significantly increased StAR levels and decreased serum testosterone levels at a high dose (250.0 mg/kg) in prenatally exposed mouse testes [30]. A low dose (128.0 µg/mL) of 27.6 ± 3.0 nm cubic crystal cerium oxide nanoparticles (CeO_2_NPs) significantly increased testosterone synthesis and upregulated the testosterone synthase gene levels in vitro [35]. A low dose (2.0 μg/mL) of 75.0 nm spherical ZnO NPs significantly increased testosterone concentration and the level of *StAR* and *CYP11A1* genes in mouse Leydig cells [36]. Numerous studies found that nanoparticles can affect steroidogenesis, and SNPs may have a similar effect on testosterone synthesis. To further determine the effect of SNPs on testosterone secretion, we demonstrated that low doses of SNPs enhanced testosterone secretion, and high doses inhibited testosterone secretion in vitro (Figure 5). Testosterone synthesis is regulated by numerous steroidogenic enzymes [37]. Among them, StAR transfers cholesterol to the mitochondrion, which is the rate-limiting step in the steroidogenesis [38]. CYP11A1 converts cholesterol to pregnenolone, and HSD3B2 transfers pregnenolone to the smooth endoplasmic reticulum (ER) and converts it to progesterone [37]. To determine whether SNPs enhanced testosterone secretion via the regulation of steroidogenic enzymes, the levels of StAR, CYP11A1, and HSD3B2 were detected. Consistent with previous studies, we found that StAR was significantly increased after low doses of SNP exposure. These results demonstrate that SNPs interfere with the normal function of Leydig cells by changing the expression pattern of testosterone biosynthesis-related enzymes at non-cytotoxic doses. Although our preliminary results were significant, a number of further studies are required to elucidate the mechanisms of SNP-regulated testosterone secretion.

Autophagy is a self-eating process that can degrade dysfunctional organelles or unfolded and misfolded proteins to provide energy and building materials to maintain cellular homeostasis [39]. In this study, we found that SNPs activated autophagy via the upregulation of BENCLIN-1 and LC3-II levels (Figure 4). LC3-II is characterized as a marker for autophagic flux measurement, which participates in autophagosome formation by converting LC3-I into LC3-II [39,40,41]. We also found that low doses of SNPs decreased P62 levels in the short term but increased them in the long term (Figure 4). P62 was found to directly bind to LC3 and was degraded by autolysosome. Once autophagy is inhibited, P62 increases, which is used as a marker for autophagic flux blocking. These results indicate that low doses of SNPs activate autophagy in the short term and cause autophagic dysfunction in the long term in PLCs. To determine the activation of autophagy by SNPs, an autophagy activator RAP, as well as inhibitors 3-MA and CQ, were used. Pre-exposure to RAP significantly enhanced autophagy, whereas pre-exposure to 3-MA or CQ significantly inhibited autophagy combined with SNPs (Figure 6). To further ensure SNP-induced autophagy, we constructed BECLIN-1 shRNA lentivirus vectors and transduced them into PLCs. BECLIN-1, used as a marker protein for autophagy formation, induces the formation of autophagosome membrane during autophagy [42]. These BECLIN-1 recombinant lentiviruses decreased BECLIN-1 levels and inhibited autophagy after SNP exposure (Figure 7). Similarly to our results, numerous studies have demonstrated that SNPs induced autophagy in other cells, such as normal rat kidney cells [43], mice spermatocyte cells [15], RAW264.7 macrophages [24,25], and human umbilical vein endothelial cells [23]. Collectively, the above evidence shows that SNPs can activate autophagy in PLCs.

Regarding the relationship between SNP-activated autophagy and testosterone secretion, pre-exposure to RAP significantly enhanced testosterone secretion, whereas pre-exposure to 3-MA or CQ significantly inhibited testosterone secretion combined with SNPs (Figure 6). Furthermore, BECLIN-1 depletion inhibited testosterone secretion after SNP and HCG exposure (Figure 7). These results indicate that at the dose range of 50–100 µg/mL SNP-activated autophagy enhances testosterone secretion in PLCs in vitro. Autophagy was extremely activated in Leydig cells and participated in testosterone production [18]. During differentiation of Leydig cells, autophagy is gradually activated from the progenitor period to the adult period and is attenuated in the aged period [44]. Yang et al. found that RAP increased testosterone in breeding and non-breeding naked mole-rat PLCs, whereas 3-MA showed opposite effects [45]. CQ has side effects on male steroid homeostasis and the structural integrity of the testes [46,47]. All these studies further demonstrate that SNPs enhance testosterone secretion via the activation of autophagy.

**Figure 8:**
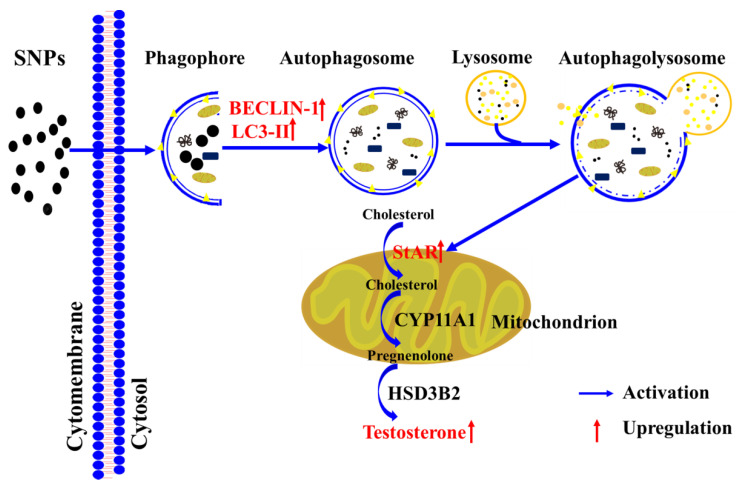
Schematic diagram of the signaling pathway involved in SNP-induced testosterone secretion in Leydig cells.

Autophagy contributes to cholesterol uptake to enhance testosterone secretion in Leydig cells [48], and StAR transfers cholesterol to the mitochondrion [38]. Combined with our results indicating that SNPs cause an increase in StAR levels, we speculated that SNP-induced autophagy would enhance testosterone secretion via the regulation of StAR levels. In this study, we found that pre-exposure to RAP significantly increased StAR levels, whereas pre-exposure to 3-MA or CQ significantly decreased StAR combined with SNPs (Figure 6). Furthermore, BECLIN-1 depletion also decreased StAR after SNP and HCG exposure (Figure 7). These results indicate that SNP-activated autophagy enhances testosterone secretion via the regulation of StAR in PLCs. A diagram of the mechanisms involved in SNP-induced Leydig cell testosterone secretion summarizes these findings (Figure 8).

Although we have demonstrated that low doses of SNPs can enhance in vitro steroidogenesis in Leydig cells, whether the activation of autophagy and the enhancement of testosterone secretion participate in the toxicity of SNPs is unclear. Additional in vivo studies should be conducted to further elucidate this. This study suggests that there is an optimum addition of SNPs, which may have no significantly negative effects on PLCs below the optimum addition level. Necessary safety precautions and exposure limits need to be specifically and separately developed for application in humans.

## 4. Materials and Methods

### 4.1. Reagents

Absolute ethanol (200 proof) and 95% ethanol were obtained from Decon Labs, Inc. (King of Prussia, PA, USA). Ammonium hydroxide (NH4OH, 28.0–30.0%), Dulbecco’s Modified Eagle’s and Ham’s F12 (DMEM/F12), and fetal bovine serum (FBS) were obtained from Fisher Scientific, Inc. (Pittsburgh, PA, USA). CCK-8 and enhanced chemiluminescence (ECL) reagents were obtained from New Cell & Molecular Biotech Co., Ltd. (Suzhou, Jiangsu, China). Annexin V-FITC/PI apoptosis detection kit, total protein extraction kit, and BCA protein assay kit were obtained from Nanjing Keygen Biotech Co., Ltd. (Nanjing, Jiangsu, China). Rabbit anti-β-actin antibody (AC026; 1:50000), rabbit anti-StAR antibody (A16432; 1:500), rabbit anti-HSD3B2 antibody (A1823; 1:500), rabbit anti-SQSTM1/P62 antibody (A19700; 1:500), and rabbit anti-BECLIN-1 antibody (A11761; 1:500) were obtained from ABclonal Inc. (Wuhan, Hubei, China). Rabbit anti-CYP11A1 antibody (orb156513; 1:1000) was obtained from Biorbyt Ltd. (Cambridge, UK). Antibody for HRP-linked anti-rabbit (7074; 1:2000) and anti-mouse (7076; 1:2000) secondary antibodies were obtained from Cell Signaling Technology (Danvers, MA, USA). Tetraethyl orthosilicate (TEOS, ≥99.0% GC), rabbit anti-LC3-II antibody (L7543; 1:1000), rapamycin (Rap; V900930), 3-methyladenine (3-MA; M9281), and chloroquine (CQ; C6628) were obtained from Sigma Aldrich Chemical Co. (St. Louis, MO, USA).

### 4.2. Animals

All male mice (Strain: C57BL/6; about 2.5 months old) were obtained from the Comparative Medicine Centre of Yangzhou University (certificate number of experimental animal production: SCXK2017-0044, Yangzhou, Jiangsu, China). The adult mice were raised in under controlled conditions of relatively constant temperature (23 ± 2 °C) and light (12 h light and 12 h darkness) and provided standard food and distilled water ad libitum. Related experimental procedures were approved by the Animal Care and Use Committee of Yangzhou University (Approval ID: 202103322).

### 4.3. Synthesis of SNPs

Spherical 100 nm Stöber silica nanoparticles were synthesized at the University of Utah as described previously [49]. Briefly, 1700 mmol of absolute ethanol, 155 mmol of DI water, and 26 mmol of ammonium hydroxide were mixed in a 250 mL flask under a stirring rate of 400 rpm for 10 min. Then, 16 mmol of TEOS was added, and the reaction was left under stirring for 24 h at room temperature. The synthesized SNPs were pelleted by centrifugation using an Avanti J-15R centrifuge (Beckman Coulter Inc., Indianapolis, IN, USA) at 11,000× *g* for 20 min and washed with distilled water and 95% ethanol three times each before being suspended in absolute ethanol for storage at a concentration of 4 mg/mL. Before use for experiments, the stock SNPs were collected by centrifugation at 20,000 rpm for 10 min, resuspended in distilled water, and dispersed using a sonicator (160 W, 20 kHz, 2 h; Bioruptor UCD-200, Belgium).

### 4.4. Characterization of SNPs

TEM (Tecnai 12; Royal Philips, Amsterdam, The Netherlands) was performed to characterize the morphology and size of the SNPs. More than 700 particles were randomly counted and measured to determine the average diameter using Image J software (National Institutes of Health, Bethesda, MD, USA). Hydrodynamic diameter and zeta potential were determined by dynamic light scattering (DLS) using a Zetasizer Nano ZS (Malvern Instruments Ltd., Worcestershire, UK).

### 4.5. Mouse Primary Leydig Cell (PLC) Culture In Vitro

After one week of adaptation to laboratory conditions, the adult mice were used to isolate PLCs following the procedure of our recent study [50]. Specifically, the testes were excised, collected, and trypsinized with collagenase I (1 mg/mL) at 37 °C for 10 min. After brief filtration and centrifugation, PLCs were resuspended and purified using discontinuous four-layer Percoll gradients (GE Healthcare Life Sciences, Piscataway, NJ, USA). The enriched PLC fraction was harvested, washed, and filtered, and the cell viability was determined by trypan blue staining. Next, the purified PLCs were centrifuged and resuspended in DMEM/F12 medium supplemented with 10% FBS and 1% penicillin/streptomycin, distributed into 6-well culture dishes, and cultured in a humidified 37 °C and 5% CO_2_ incubator. The purity of PLCs was assessed by 3β-hydroxysteroid dehydrogenase type II (HSD3B2), and about 95% of the cells were identified as Leydig cells using HSD3B2 staining [28].

### 4.6. Detection of the Cellular Internalization of SNPs in PLCs

PLCs were exposed to 100 μg/mL of SNPs suspended in DMEM/F12 and 10% FBS for 24 h. Then, the cells were washed with PBS three times to remove the excess SNPs, trypsinized, and centrifuged. Next, PLC pellets were fixed in 2.5% glutaraldehyde for 2 h at room temperature. After washing with PBS three times, the cells were embedded in 2% agarose gel, postfixed in 4% osmium tetroxide solution, stained with 0.5% uranyl acetate, dehydrated through a graded ethanol series, and embedded in epoxy resin. The samples were polymerized at 60 °C for 48 h. Finally, ultrathin sections were stained with 5% aqueous uranyl acetate and 2% aqueous lead citrate, air dried, and imaged by TEM (Tecnai 12; Royal Philips, Amsterdam, The Netherlands).

### 4.7. Determination of Cell Viability

The effect of SNPs on cell viability was determined using a CCK-8 kit. PLCs were cultured in 6-well culture dishes at a density of 2 × 10^6^ cells/well. After 24 h, PLCs were trypsinized, centrifuged, resuspended, and seeded into 96-well plates at a density of 3 × 10^4^ cells/200 µL medium/well for 24 h. Next, the cells were treated with fresh DMEM/F12 medium containing 0, 50, 100, 200, 300, 400, 500, 600, 800, and 1000 µg/mL SNPs for 24 h, and subsequently, the CCK-8 reagent was added and incubated for 2 h. The absorbance at 450 nm was measured using a microplate reader (Model 680, Bio-Rad, Hercules, CA, USA). Experiments were repeated independently in triplicate.

### 4.8. Measurement of Cell Apoptosis

PLCs were cultured in 6-well culture dishes at a density of 1 × 10^6^ cells/well. After 24 h, PLCs were treated with 0, 50, 100, and 200 µg/mL SNPs and subsequently incubated for another 24 h. Next, PLCs were trypsinized, collected, and quantified with a cell apoptosis detection kit (Nanjing KeyGen Biotech, Nanjing, Jiangsu, China). According to the manufacturer’s instructions, the cells were stained with 5 µL of Annexin V-FITC at room temperature for 15 min in the dark, and then 400 μL of binding buffer containing 5 μL of propidium iodide (PI) was added and incubated for another 15 min. The apoptotic rate was detected using flow cytometry (CytoFLEX S, Beckman Coulter, Inc., Brea, CA, USA). Experiments were repeated independently in triplicate.

### 4.9. Monodansylcadaverine (MDC) Staining

SNP-induced autophagy was monitored using an MDC detection kit (Nanjing KeyGen Biotech, Nanjing, Jiangsu, China). PLCs were cultured on sterile cover slips placed in 24-well culture dishes for 24 h and then treated with 0, 50, 100, and 200 µg/mL SNPs and subsequently incubated for another 24 h. After washing with PBS three times, the cells were incubated with MDC at room temperature for 45 min in the dark and then washed again with PBS three times. Finally, the cells were examined under a laser scanning confocal microscope (TCS SP8 STED; Wetzlar, Hessen, GER).

### 4.10. Measurement of Testosterone Secretion

PLCs were cultured in 6-well culture dishes at a density of 1 × 10^6^ cells/well. After 24 h, PLCs were treated with 0, 50, 100, and 200 µg/mL SNPs in the presence of 1 IU/mL HCG. Next, the culture supernatants were harvested, and testosterone secretion was measured using a testosterone detection kit (Shanghai Hufeng Biotechnology Co., Ltd., Shanghai, China) via enzyme-linked immunosorbent assay (ELISA) according to the manufacturer’s instructions. The absorbance at 450 nm was measured using a microplate reader (Model 680, Bio-Rad, Hercules, CA, USA). Experiments were repeated independently in triplicate.

### 4.11. BECLIN-1 Short Hairpin Interfering RNA (shRNAs) Lentivirus Transduction

BECLIN-1 shRNA recombinant lentivirus vectors (shBec-1 and shBecl-2) and a negative control shRNA vector (shNC) were constructed. The sequences of the shRNAs are listed in Table 1. The process of lentivirus packaging was reported in our previous study [50]. Briefly, recombinant lentivirus vectors and packaging vectors (pGag/Pol, pRev, and pVSV-G) were co-transfected into HEK 293T cells. After transfection for 48 h, the lentivirus supernatants were harvested, purified, filtered, concentrated, and stored at −80 °C. PLCs were transduced with an appropriate number of lentiviral particles (multiplicity of infection (MOI) = 20) containing 8 µg/mL polybrene in DMEM/F12. After transduction for 12 h, the lentivirus-containing medium was removed and replaced with fresh DMEM/F12 for another 48 h. Next, the cells were exposed to 100 µg/mL SNPs for 12 h. Finally, the cells and supernatants were harvested for testosterone measurement.

### 4.12. Real-Time Quantitative PCR (RT-qPCR)

The process of RT-qPCR was reported in our previous study [51]. Briefly, total RNA was isolated from PLCs using an RNA extraction reagent kit (TaKaRa Bio, Inc., Dalian, China). cDNA was synthesized using a reverse transcription reagent kit (TaKaRa Bio, Inc., Dalian, China). RT-qPCR was performed with a Roche Light Cycler 480 II PCR instrument using a SYBR premix kit (TaKaRa Bio, Inc., Dalian, China) according to the manufacturer’s instructions. The sequences of the specific primers are listed in Table 2. Each gene was repeated three times as technical replicates. The 2^−ΔΔCt^ method was used to perform the gene mRNA quantifications, and β-actin was used as an internal control gene to normalize the amount of transcripts in each sample to correct for differences in the cDNAs used.

### 4.13. Western Blotting

PLCs were trypsinized and collected, and total cellular proteins were extracted using a protein extraction kit. A bicinchoninic acid (BCA) protein assay kit was used to measure the protein concentration. Equal amounts of total proteins (20 µg) were separated with 12% sodium dodecyl sulfate-polyacrylamide gel electrophoresis (SDS-PAGE). The proteins on the gels were transferred to 0.22 μm polyvinylidene difluoride (PVDF) membranes (Millipore, Bedford, MA, USA), blocked with 10% skim milk in Tris-buffered saline (TBS) for 1 h at room temperature, and subsequently incubated with different primary antibodies at 4 °C. After 12 h, the membranes were incubated with the HRP-labeled secondary antibodies at room temperature for 1 h. Immunoreactive bands were scanned using a gel imaging system (Tannon Science & Technology, Shanghai, China). Protein quantification was analyzed with Quantity One software (Bio-Rad, Hercules, CA, USA). Experiments were repeated independently in triplicate.

### 4.14. Statistical Analysis

All of the data are presented as mean ± SEM. All of the experiments were repeated independently, at least in triplicate. One-way ANOVA, followed by Fisher’s least significant different test (Fisher LSD) and an independent samples *t* test with the Statistical Package for the Social Sciences (SPSS) software (Version 18.0, Chicago, IL, USA), was used to analyze the data. The critical value for statistical significance was *p* < 0.05.

## 5. Conclusions

In summary, this study demonstrated that low doses of SNPs increased testosterone secretion via the increase in StAR levels and activated autophagy in PLCs. Furthermore, SNP-activated autophagy can enhance testosterone secretion and StAR levels. Taken together, our experiments indicate that SNPs can enhance testosterone secretion via the activation of autophagy in PLCs.

## Figures and Tables

**Table 1:** Sequences of shRNAs.

Interference Fragment	Synthetic Primer	Sequence (5′–3′)
shBec-1	shBec-1F	GATCCCAGTCTCTGACAGACAAATCTCTCGAGAGATTTGTCTGTCAGAGACTGTTTTTG
	shBec-1R	AATTCAAAAACAGTCTCTGACAGACAAATCTCTCGAGAGATTTGTCTGTCAGAGACTGG
shBec-2	shBec-2F	GATCCCAATAAGATGGGTCTGAAGTTCTCGAGAACTTCAGACCCATCTTATTGTTTTTG
	shBec-2R	AATTCAAAAACAATAAGATGGGTCTGAAGTTCTCGAGAACTTCAGACCCATCTTATTGG
shNC	shNC-F	GATCCGATGAAATGGGTAAGTACACTCGAGTGTACTTACCCATTTCATCTTTTTG
	shNC-R	AATTCAAAAAGATGAAATGGGTAAGTACACTCGAGTGTACTTACCCATTTCATCG

**Table 2:** Sequences of qPCR.

Genes	GenBank Number	Forward Primers (5′–3′)	Reverse Primers (5′–3′)	Product Size (bp)
*β-actin*	NM_007393.5	GCAAGCAGGAGTACGATGAG	CCATGCCAATGTTGTCTCTT	148
*BECLIN-1*	NM_019584.3	ATGGAGGGGTCTAAGGCGTC	TCCTCTCCTGAGTTAGCCTCT	197
*LC3-II*	NM_026160.4	CGGCTTCCTGTACATGGTTT	AACCATTGGCTTTGTTGGAG	83
*P62*	NM_001290769.1	ACAGCCAGAGGAACAGATGG	GGAGGGTGCTTCGAATACTG	240
*StAR*	NM_011485.4	CTTGGCTGCTCAGTATTGAC	TGGTGGACAGTCCTTAACAC	153
*CYP11A1*	NM_019779.3	CGATACTCTTCTCATGCGAG	CTTTCTTCCAGGCATCTGAAC	126
*HSD3B2*	NM_153193.3	CATTCCTGCTGGAAACTGTGAGC	ATCTCGCTGAGCTTTCTTGTAGG	128

## Data Availability

The data sets used and analyzed during the current study are available from the corresponding authors on request.

## Supplementary Materials

The following are available online at https://www.mdpi.com/article/10.3390/ijms23063104/s1.
