# Supplementary material for: Activation of Autophagy by Low-Dose Silica Nanoparticles Enhances Testosterone Secretion in Leydig Cells
Source: Int J Mol Sci. 2022 Mar 13;23(6):3104. doi: 10.3390/ijms23063104 (PMC8949068; doi:10.3390/ijms23063104)
Supplement: Supplementary file 1 [file ijms-23-03104-s001.zip › ijms-1624864-supplementary.pdf]

# Supplementary material

# Activation of Autophagy by Low-Dose Silica Nanoparticles Enhances Testosterone Secretion in Leydig Cells

**Jinlong Zhang [1,2,3], Rongrong Ye [1,2], Jason William Grunberger [4,5], Jiaqi Jin [1,2], Qianru Zhang [1,2], Raziye Mohammadpour [4,5], Nitish Khurana [4,5], Xianyu Xu [1,2,3], Hamidreza Ghandehari [4,5,6] and Fenglei Chen [1,2,3,*]**

1 College of Veterinary Medicine, Yangzhou University, Yangzhou 225009, China; zjl@yzu.edu.cn (J.Z.); yrr971106@163.com (R.Y.); savannahkini@163.com (J.J.); longlongzhang12345@126.com (Q.Z.); xuxianyu@yzu.edu.cn (X.X.)
2 Jiangsu Co-innovation Center for Prevention and Control of Important Animal Infectious Diseases and Zoonoses, Yangzhou 225009, China
3 Joint International Research Laboratory of Agriculture and Agri-Product Safety of the Ministry of Education of China, Yangzhou University, Yangzhou 225009, China
4 Department of Pharmaceutics and Pharmaceutical Chemistry, University of Utah, Salt Lake City, UT 84112, USA; u1204188@utah.edu (J.W.G.); raziye.mohammadpour@utah.edu (R.M.); nitish.khurana@utah.edu (N.K.); hamid.ghandehari@utah.edu (H.G.)
5 Utah Center for Nanomedicine, University of Utah, Salt Lake City, UT 84112, USA
6 Department of Biomedical Engineering, University of Utah, Salt Lake City, UT 84112, USA
* Correspondence: flchen@yzu.edu.cn; Tel: +86-514-87979030; Fax: +86-514-87972218

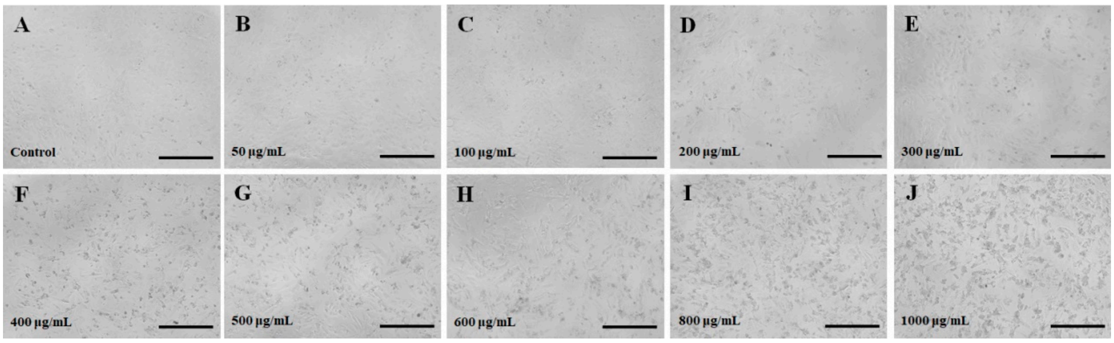


**Figure S1.** Effect of SNPs on cell morphology in PLCs. Cells (**A–J**) were exposed to 0, 50, 100, 200, 300, 400, 500, 600, 800, and 1000 μg/mL SNPs for 24 h, respectively. Cell morphology was recorded and compared by digital microscopy (BA400, Motic, Amoy, China). Scale bars, 100 μm.
